# Thermal Shock Behavior of Si_3_N_4_/BN Fibrous Monolithic Ceramics

**DOI:** 10.3390/ma16196377

**Published:** 2023-09-24

**Authors:** Qingqing Chen, Yuan Zhang, Yu Zhou, Daxin Li, Guobing Ying

**Affiliations:** 1 Department of Materials Science and Engineering, College of Mechanics and Materials, Hohai University, Nanjing 211100, China; zhangyuan795@163.com; 2 Institute for Advanced Ceramics, School of Materials Science and Engineering, Harbin Institute of Technology, Harbin 150001, China; zhouyu@hit.edu.cn (Y.Z.); lidaxin@hit.edu.cn (D.L.); 3 Key Laboratory of Advanced Structural-Functional Integration Materials and Green Manufacturing Technology, Harbin Institute of Technology, Harbin 150001, China; 4 School of Materials Science and Engineering, Harbin Institute of Technology (Shenzhen), Shenzhen 518055, China

**Keywords:** Si3N4/BN fibrous monolithic ceramics, thermal shock behavior, mechanical

## Abstract

To develop materials suitable for aerospace applications, silicon nitride/boron nitride (Si${}_{3}$N${}_{4}$/BN) fibrous monolithic ceramics with varying BN contents were prepared. Employing analytical techniques such as XRD and SEM, coupled with mechanical testing equipment, the influence of BN concentration on the thermal shock resistance of Si${}_{3}$N${}_{4}$/BN fibrous monolithic ceramics was assessed. When the thermal shock differential is less than 800 °C, its residual flexural strength gradually decreases as the thermal shock differential increases. Conversely, when the differential exceeds 1000 °C, the residual flexural strength of the material increases. The residual strength of all samples reached its peak after undergoing a thermal shock assessment at a 1500 °C differential. When the BN mass fraction is 5 wt.%, the residual strength after a thermal shock at a temperature difference of 1500 °C is 387 ± 19 MPa, which is 124% higher than the original strength of the sample that did not undergo thermal shock (25 °C, 311 ± 18 MPa). The oxide layer formed on the thermal shock surface played a role in bridging defects introduced during material surface processing.

## 1. Introduction

In the face of rising challenges from aerospace superpowers and China’s evolving defense industry, it is crucial to maintain the structural integrity of hypersonic vehicles under extreme conditions. Hypersonic vehicles require improved anti-ablative and thermal insulation materials, especially in the nose cone and leading edge, to endure speeds exceeding 5 Mach, durations beyond 15 min, and temperatures over 2000 °C [1]. Anti-oxidation materials are also essential, with exacting standards for temperature endurance, durability, and performance under high-temperature oxidation and complex loads [2,3,4,5,6].

Traditional ceramics, employed in radomes, present various challenges. For example, the high thermal expansion coefficient of alumina ceramics results in inadequate thermal shock resistance. The limited application scope of crystalline glass is due to its low melting point and significant dielectric loss at elevated temperatures. Quartz glass and BN ceramics exhibit compromised mechanical strengths, increased susceptibility to moisture, and decreased erosion resistance. Silicon nitride (Si${}_{3}$N${}_{4}$), distinguished by its hexagonal lattice structure, excels in high-temperature oxidation resistance, mechanical robustness, thermal expansion control, and thermal shock resilience. Despite their superior attributes, Si${}_{3}$N${}_{4}$ ceramics are hindered by limited machinability and thermal shock resistance. Blending boron nitride (BN), which boasts a decomposition threshold of 3000 °C and improved thermal attributes, with Si${}_{3}$N${}_{4}$ may overcome these limitations [7,8].

Presently, Si${}_{3}$N${}_{4}$-BN composite ceramics derive from methodologies such as atmospheric pressure sintering, reaction sintering, hot pressing sintering, and spark plasma sintering (SPS) [9,10,11,12,13,14]. Though atmospheric pressure and reaction sintering yield ceramics with compromised densities and defect ratios, spark plasma sintering, albeit efficient, is constrained by its infancy stage, complexity, and prohibitive costs. Meanwhile, hot pressing emerges as a versatile, cost-efficient method ensuring optimal product density and performance. Employing axial pressure during the heating phase optimizes the raw powder interaction, facilitating superior composite synthesis [15,16,17,18,19,20]. Drawing inspiration from natural biomaterials, the research initiatives have replicated nacre’s layered structure to engineer high-resilience biomimetic ceramics. Such layering deflects cracks, enhancing energy absorption and fortifying ceramic resilience. Noteworthy developments by Kovar have integrated Si${}_{3}$N${}_{4}$ fibers to produce Si${}_{3}$N${}_{4}$/BN fibrous monolithic ceramics, achieving flexural strengths of approximately 450 MPa [21,22]. However, the thermal shock resistance data for Si${}_{3}$N${}_{4}$/BN fibrous monolithic ceramics remains scarce.

Given that ceramics frequently experience thermal shocks caused by drastic temperature fluctuations during service, their thermal shock resistance reflects a comprehensive manifestation of the material’s mechanical and thermal properties. This paper evaluates the thermal shock resistance of materials by measuring the residual flexural strength of the material before and after thermal shock. The study assesses the residual flexural strength, phase composition, and microscopic morphology changes of Si${}_{3}$N${}_{4}$/BN fibrous monolithic ceramic materials with varying BN content. It examines the impact of BN phase content and thermal shock temperature differences on the thermal shock resistance of Si${}_{3}$N${}_{4}$/BN fibrous monolithic ceramic materials and the underlying damage mechanisms.

## 2. Materials and Methods

### 2.1. Raw Materials

**Figure 1:**
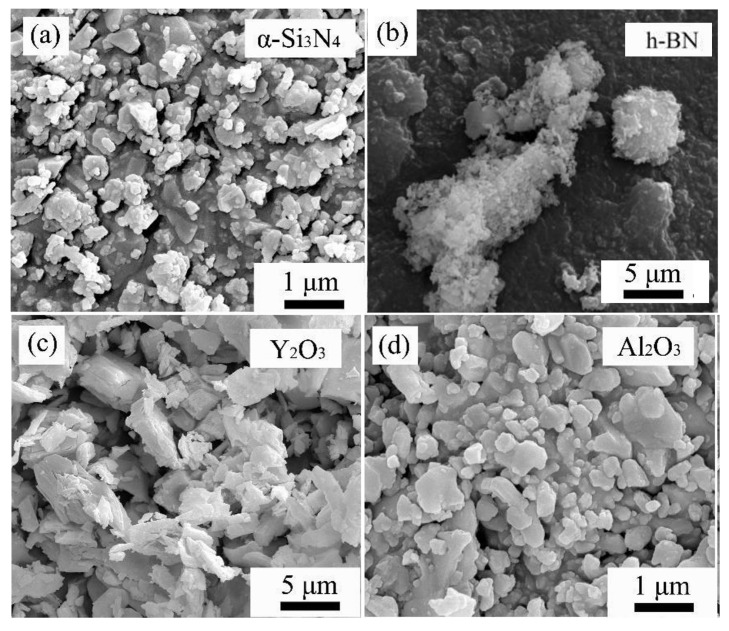
SEM morphologies of the raw powders: (**a**) α-Si${}_{3}$N${}_{4}$. (**b**) BN. (**c**) Y${}_{2}$O${}_{3}$. (**d**) Al${}_{2}$O${}_{3}$.

**Figure 2:**
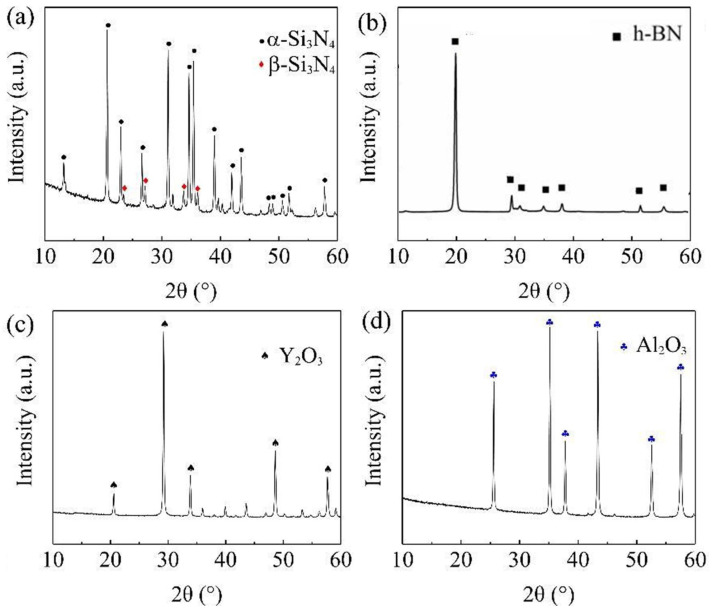
XRD patterns of the raw powders: (**a**) α-Si${}_{3}$N${}_{4}$. (**b**) BN. (**c**) Y${}_{2}$O${}_{3}$. (**d**) Al${}_{2}$O${}_{3}$.

The powders used in this experiment are α-Si${}_{3}$N${}_{4}$, BN, yttrium(III) oxide (Y${}_{2}$O${}_{3}$), and aluminum oxide (Al${}_{2}$O${}_{3}$). Figure 1 and Figure 2 are the SEM images and XRD spectra of the raw material powders, respectively. From the surface morphology, it can be observed that the raw material size distribution is uniform, and the size meets the requirements. From the X-ray diffraction spectrum, it is evident that the purchased α-Si${}_{3}$N${}_{4}$ and h-BN powders have high purity. The Si${}_{3}$N${}_{4}$ powder contains both α-Si${}_{3}$N${}_{4}$ and β-Si${}_{3}$N${}_{4}$ phases, with the α phase being dominant and a minor amount of the β phase.

### 2.2. Experimental Procedure

**Figure 3:**
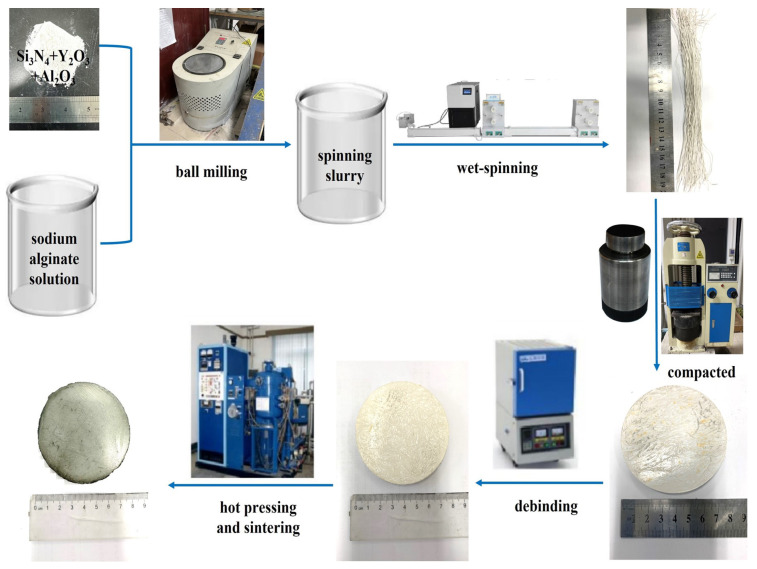
The process flowchart of Si${}_{3}$N${}_{4}$/BN fibrous monolithic ceramics.

The preparation method for Si${}_{3}$N${}_{4}$/BN fibrous monolithic ceramic is illustrated in Figure 3. A sodium alginate solution is mixed uniformly with the initial powder to obtain a spinning slurry (for the specific ratios, refer to Table 1). A designated amount of BN powder is dispersed in deionized water, creating four different weight fractions of BN slurries (Table 2). The Si${}_{3}$N${}_{4}$ fibers are then extruded using a wet-spinning device and coated with BN. After air drying at room temperature, the fibers are trimmed to a specified size, aligned in a chromium steel mold, and pre-pressed at 20 MPa for 5 min, yielding Si${}_{3}$N${}_{4}$/BN fibrous monolithic ceramic green bodies. Once the green bodies are dried at 60 °C, they are heated in a muffle furnace at a rate of 1 °C/min up to 600 °C and held for 1 h for debinding. Under a 0.1 MPa N${}_{2}$ atmosphere and a uniaxial pressure of 20 MPa, the equipment is heated at 15 °C/min to 1800 °C. After maintaining this temperature for 2 h, the furnace cools, producing pure Si${}_{3}$N${}_{4}$/BN fibrous monolithic ceramics. Post-sintering, the ceramic samples are removed from the mold. Using in-house equipment such as a surface grinder, internal and external circular cutters, and an external grinder, the samples are processed into dimensions of 3 × 4 × 36 mm and 2 × 4 × 22 mm in preparation for subsequent mechanical and thermal shock resistance testing.

### 2.3. Characterization

The characterization of the bulk samples was conducted using X-ray diffraction (XRD) (Rigaku Corporation, Tokyo, Japan). The equipment utilized for the XRD test was the D/max-γB type X-ray diffractometer from Rigaku Corporation (Tokyo, Japan). The microstructure of the material is characterized by imaging with a scanning electron microscope (SEM, including XL 30, TM 3000, HITACHI S4800, and Sirion 2000, Hitachi, Chiyoda, Tokyo) and is analyzed using an energy dispersive spectrometer (EDS) (PhotoMetrics, Inc., Huntington Beach, CA, USA) to examine the micro-structure of Si${}_{3}$N${}_{4}$/BN fibrous monolithic ceramics.

The mechanical properties, specifically the three-point flexural strength and fracture toughness of Si${}_{3}$N${}_{4}$/BN fibrous monolithic ceramics, were evaluated at room temperature using the INSTRON-5569 universal testing machine (Instron Group, Norwood, MA, USA). To prepare for this, ceramic blocks underwent a series of processes: they were cut using an internal circular cutting machine, ground, polished to achieve a mirror finish, chamfered, and cleaned. This resulted in test samples of dimensions 3 × 4 × 36 mm and 2 × 4 × 22 mm.

For the flexural strength measurement, 3 × 4 × 36 mm specimens were selected, following the GB/T1965-1996 [23] standard. The tests were conducted with a 30 mm span and a loading speed of 0.5 mm/min. The results from 4–6 samples were averaged. The flexural strength was calculated using Formula (1):

(1) $$\sigma =\frac{3PL}{2BW^{2}}$$

where σ represents the flexural strength (MPa); *P* is the maximum applied load (N); *L* is the span during testing (30 mm); *B* is the width (approximately 4 mm); and *W* is the height (approximately 3 mm).

The fracture toughness was determined following the GB/T 23806-2009 [24] standards, using 2 × 4 × 20 mm specimens and the single-edge notched beam method. The notch depth was kept under 2mm. During testing, the maximum fracture load was recorded, the notch depth was measured, and the press head’s movement rate was set at 0.05 mm/min with a 16 mm span. The results from 4–6 samples were averaged. The fracture toughness was calculated using Equation (2):

(2) $$K_{IC}=\frac{3PL\sqrt{a\times 10^{-3}}}{2BW^{2}}\left[1.93-3.07\left(\frac{a}{W}\right)+14.53{\left(\frac{a}{W}\right)}^{2}-25.11{\left(\frac{a}{W}\right)}^{3}+25.8{\left(\frac{a}{W}\right)}^{4}\right]$$

where $K_{IC}$ is the fracture toughness ( MPa·m${}^{1/2}$); *P* is the maximum applied load (N); *L* is the span (16 mm); *a* is the notch depth (mm); *B* is the width (approximately 2 mm); and *W* is the height (approximately 4 mm).

For the thermal shock resistance measurement, 3 × 4 × 36mm specimens were selected, following the GB/T 16536-1996 [25] and GB/T 10700-2006 [26] standards. Thermal shock tests were conducted in a high-temperature box-type resistance furnace set to the designated thermal shock temperature. Once the desired temperature was attained, the alumina crucible containing the thermal shock samples was quickly placed in the resistance furnace and held for 10 min. After this duration, the samples were swiftly removed and rapidly cooled in water. The temperature differentials for the thermal shock tests were set at six levels: 600 °C, 800 °C, 1000 °C, 1200 °C, 1400 °C, and 1500 °C. The residual flexural strength of the samples post-thermal shock was measured using an Instron-5569 (Instron Group, Norwood, MA, USA) universal testing machine from the USA. After gold sputtering on the sample surfaces and fracture faces, the microstructure and morphology of the post-thermal shock ceramic samples were observed under a Helios Nanolab 600i (FEI Corporation, Hillsboro, OR, USA) scanning electron microscope.

## 3. Results and Discussions

### 3.1. Mechanical Properties

**Figure 4:**
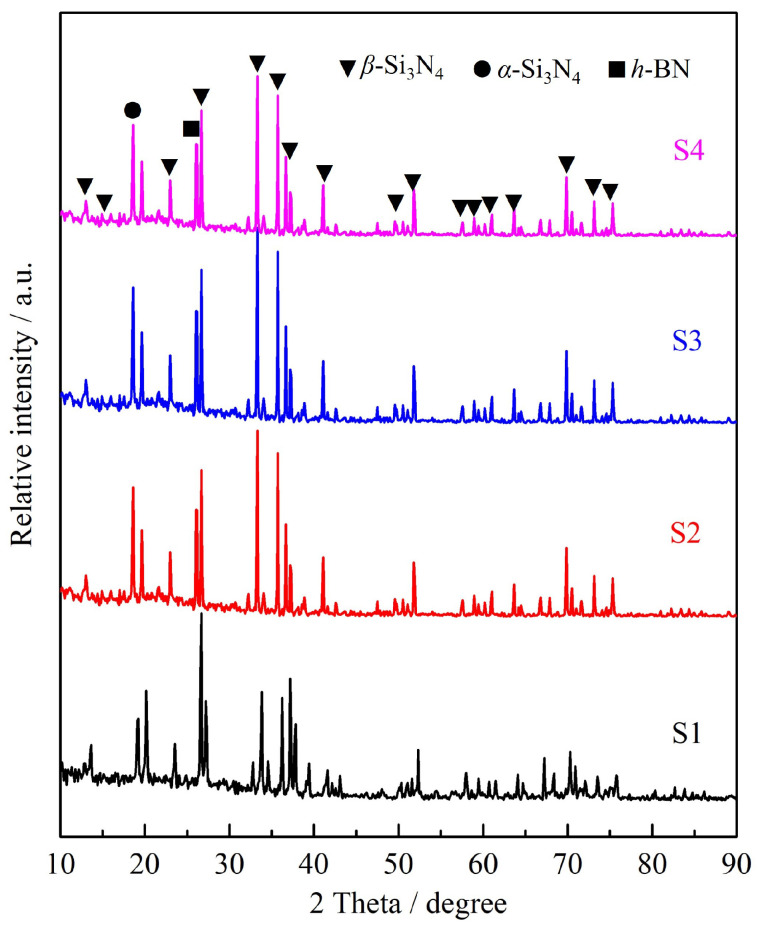
XRD patterns of Si${}_{3}$N${}_{4}$/BN fibrous monolithic ceramics with different BN contents.

Figure 4 shows the phase diffraction peaks of Si${}_{3}$N${}_{4}$/BN fibrous monolithic ceramics with different BN contents. It can be observed that the main phases of hot-pressed Si${}_{3}$N${}_{4}$/BN whisker ceramics are β-Si${}_{3}$N${}_{4}$, α-Si${}_{3}$N${}_{4}$, and h-BN. This confirms that at 1800 °C, the transformation from the α-phase to the β-phase is essentially achieved [27,28,29]. Due to the low content of the sintering aids Y${}_{2}$O${}_{3}$-Al${}_{2}$O${}_{3}$, the diffraction peak intensities of β-Si${}_{3}$N${}_{4}$ and h-BN are high, and the presence of Y${}_{2}$O${}_{3}$ and Al${}_{2}$O${}_{3}$ diffraction peaks could not be detected.

Table 3 presents the effects of BN content on the relative density and apparent porosity of Si${}_{3}$N${}_{4}$/BN fibrous monolithic ceramics. The theoretical densities of Si${}_{3}$N${}_{4}$, BN, Y${}_{2}$O${}_{3}$, and Al${}_{2}$O${}_{3}$ are 3.44 g·cm${}^{-3}$, 2.27 g·cm${}^{-3}$, 5.01 g·cm${}^{-3}$, and 3.5 g·cm${}^{-3}$, respectively [30]. Si${}_{3}$N${}_{4}$/BN fibrous monolithic ceramics with BN concentrations of 3 wt.%, 5 wt.%, 10 wt.%, and 50 wt.% were prepared through hot-pressing at 1800 °C/20 MPa/2 h/N${}_{2}$. They were labeled S1, S2, S3, and S4, with achieved relative densities of 98.3%, 96.8%, 93.5%, and 83.8%, respectively. Table 1 reveals that increasing the BN concentration leads to a decline in relative density and a rise in apparent porosity. The apparent porosity ranges from 0.93% to 3.52%, indicating that the Si${}_{3}$N${}_{4}$/BN fibrous monolithic ceramic material has a good overall densification. The growth of Si${}_{3}$N${}_{4}$ grains is mainly achieved through the dissolution–precipitation process, and the sintering aids Y${}_{2}$O${}_{3}$-Al${}_{2}$O${}_{3}$ added in this study react with the Si${}_{x}$N${}_{y}$ on the surface of Si${}_{3}$N${}_{4}$ at 1800 °C, producing a silicate liquid phase (Si-Al-Y-O-N) with a low eutectic point that wets and envelops the Si${}_{3}$N${}_{4}$ grains [31].

**Figure 5:**
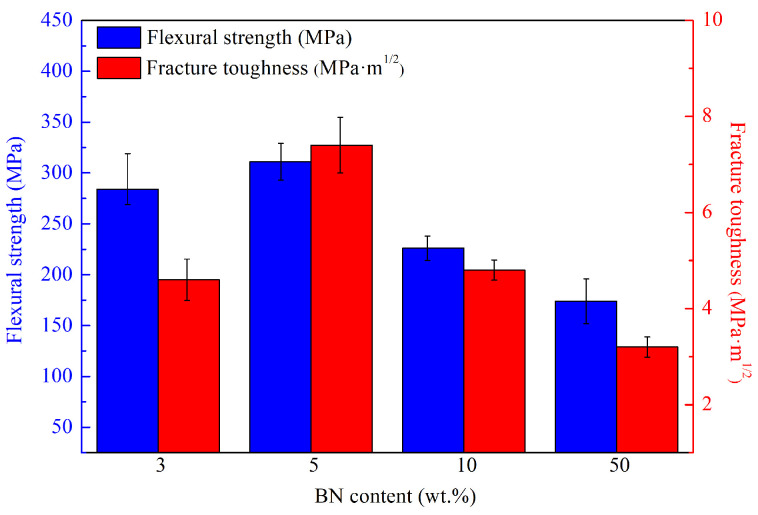
The mechanical properties of Si${}_{3}$N${}_{4}$/BN fibrous monolithic ceramics with different BN content.

Figure 5 illustrates the impact of BN content on the mechanical properties of Si${}_{3}$N${}_{4}$/BN fibrous monolithic ceramics. The data reveal that as the BN content increases from 3 wt.% to 50 wt.%, considering the error margin, the flexural strength of the 3 wt.% (S1) and 5 wt.% (S2) samples remains relatively consistent, registering at 294 ± 25 MPa and 311 ± 18 MPa, respectively. However, when the BN mass fraction rises to 10 wt.% and 50 wt.%, the flexural strength decreases to 226 ± 12 MPa and 174 ± 22 MPa, respectively. This trend underscores the role of BN in attenuating the material’s flexural strength, consistent with the changes in densification. The fracture toughness initially increases with the rising BN content and then decreases. When the BN mass fraction is 5 wt.% (S2), the fracture toughness is 7.4 ± 0.58 MPa·m${}^{1/2}$, a notable 161% surge relative to the 3 wt.% (S1). Incorporating an optimal BN quantity results in the refinement of Si${}_{3}$N${}_{4}$ grains, with β-Si${}_{3}$N${}_{4}$ grains amplifying the material’s resilience, thereby augmenting fracture toughness [31]. However, an overabundance of BN curtails the ceramic’s fracture toughness attributed to inferior mechanical properties. At a mass fraction of 50 wt.% (S4), the fracture toughness drops to its lowest at 3.2 ± 0.21 MPa·m${}^{1/2}$.

### 3.2. Residual Flexural Strength

**Figure 6:**
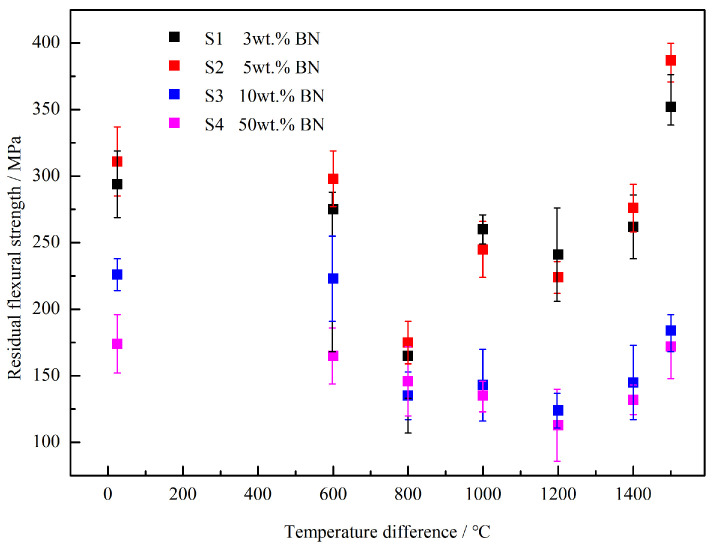
Residual flexural strength for Si${}_{3}$N${}_{4}$/BN ceramics with different BN concentrations under different thermal-shock temperature differences.

**Figure 7:**
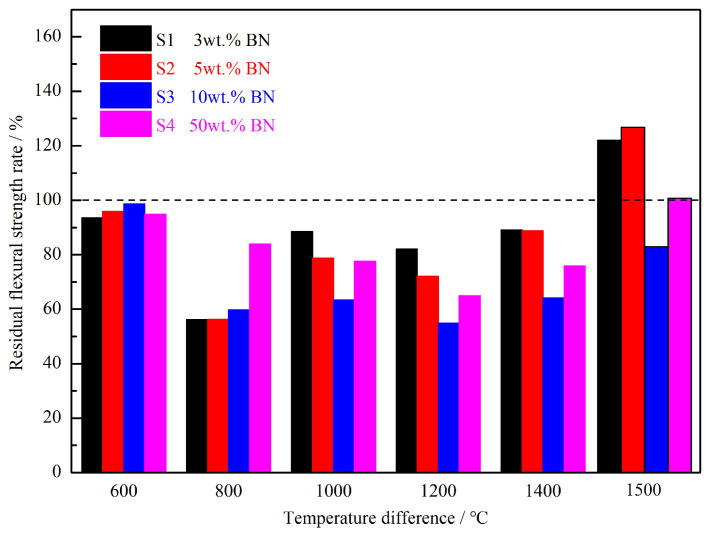
Residual flexural strength rate of Si${}_{3}$N${}_{4}$/BN fibrous monolithic ceramics with different BN concentrations under different thermal-shock temperature differences.

According to the standards GB/T 16536-1996 [25] and GB/T 10700-2006 [26], the Si3N4/BN fibrous monolithic ceramics prepared under the conditions of 1800 °C/20 MPa/2 h/N${}_{2}$ were tested for residual flexural strength (Figure 6) and residual flexural strength rate (Figure 7) under different thermal shock temperature differences using the cold quenching method. This was implemented to evaluate the thermal shock resistance of the ceramics. The thermal shock temperature differences used in this experiment were 600 °C, 800 °C, 1000 °C, 1200 °C, 1400 °C, and 1500 °C. Figure 6 shows that the samples with BN mass fractions of 3 wt.%, 5 wt.%, 10 wt.%, and 50 wt.% exhibit an overall trend of decreasing and then increasing residual flexural strength with the rise in thermal shock temperature difference. Specifically, when the thermal shock temperature difference is below 800 °C, the residual flexural strength of the material gradually decreases with the increase in thermal shock temperature difference. This is because the larger the thermal shock temperature difference, the greater the thermal shock impact on the sample, resulting in greater damage. When the thermal-shock temperature difference exceeds 1000 °C, the material’s residual flexural strength increases. After undergoing a thermal shock assessment at a temperature difference of 1500 °C, the residual strength of all samples reached its highest value. Among them, the residual flexural strength retention rates of the S1 (3 wt.%) and S2 (5 wt.%) samples were both greater than 100%, indicating that the flexural strength of the material at this time was higher than the room temperature. Notably, when the BN mass fraction is 5 wt.% (S2), the residual strength after a thermal shock at a temperature difference of 1500 °C is 387 ± 19 MPa, which is 124% higher than the original strength of the sample that did not undergo thermal shock (25 °C, 311 ± 18 MPa).

### 3.3. Surface Phase Composition

**Figure 8:**
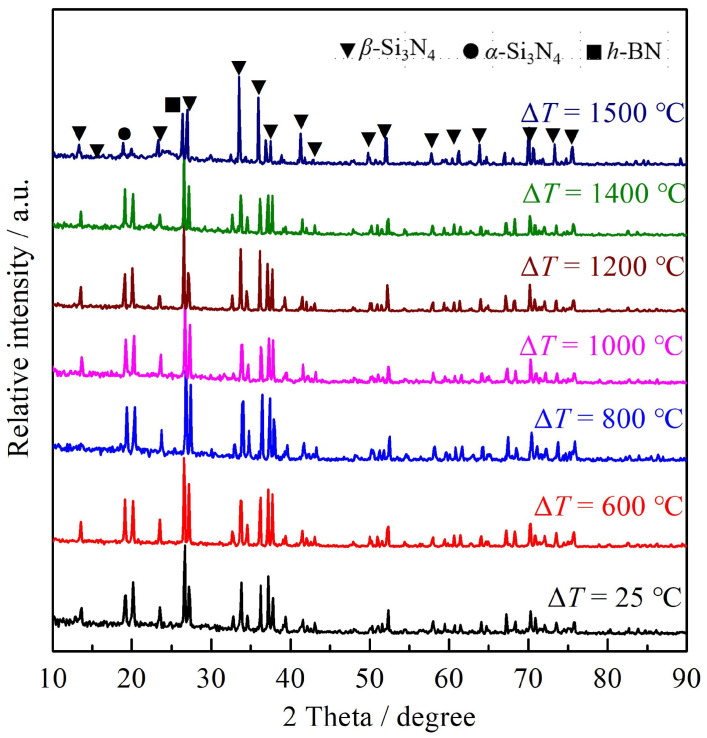
XRD of S2 sample surfaces after thermal shock at several temperature differences.

The XRD spectrum in Figure 8 illustrates the phase composition of the S2 sample surfaces post thermal shock at several temperature differences. The primary crystalline phases on the pristine sample’s surface, which has not undergone thermal shock, are β-Si${}_{3}$N${}_{4}$ and BN. In the presence of a high-temperature oxidative atmosphere during the thermal-shock process, the composite ceramic surface experiences significant physical and chemical alterations, notably oxidation and volatilization. As per the literature reviews, BN, when exposed to temperatures exceeding 450 °C, can oxidize to form the liquid phase B${}_{2}$O${}_{3}$. Furthermore, B${}_{2}$O${}_{3}$ begins to volatilize swiftly above 800 °C [32,33]. However, no traces of B${}_{2}$O${}_{3}$ were detected on the sample surface post thermal shock at a 600 °C differential. This can be attributed to the brief heat retention during the shock and the ambient presence of water vapor, which depresses B${}_{2}$O${}_{3}$’s volatilization threshold.

### 3.4. Surface Morphology

**Figure 9:**
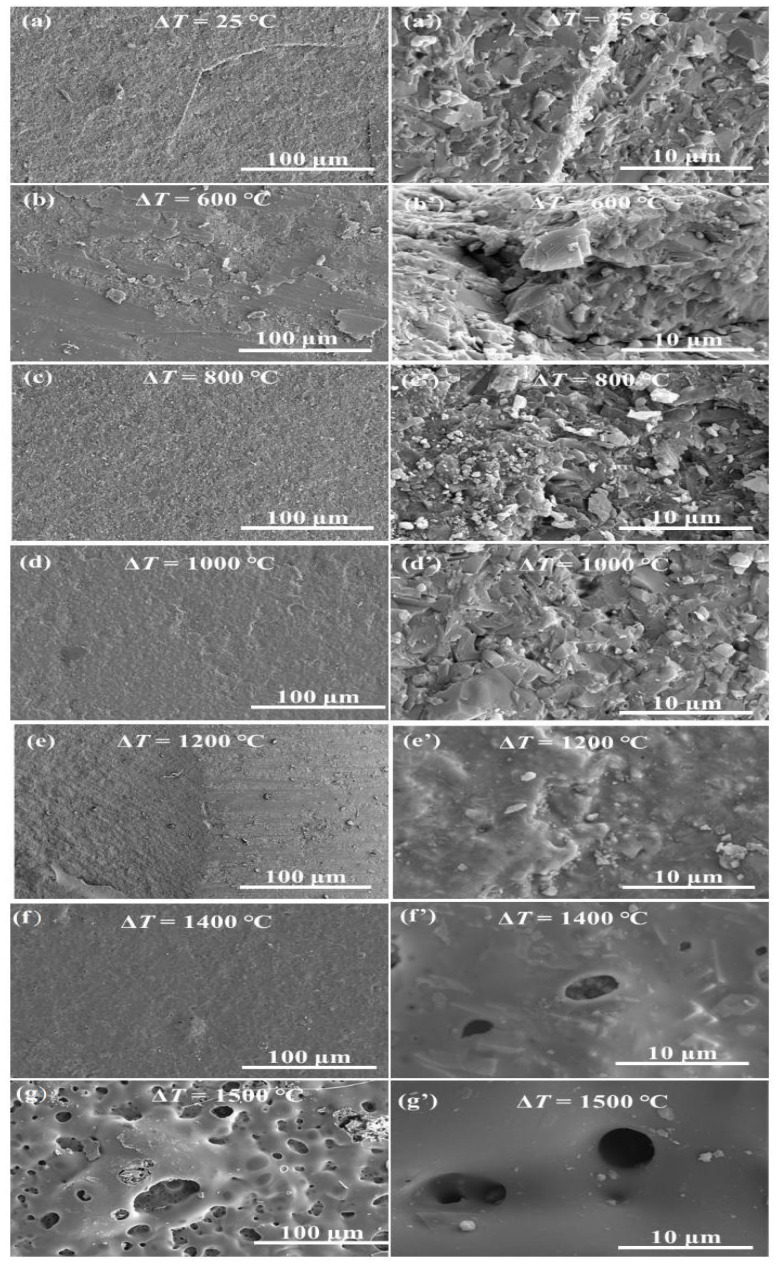
Surface micrographs of S2 samples after thermal shock at several temperature differences: (**a**,**a’**) 25 °C, (**b**,**b’**) 600 °C, (**c**,**c’**) 800 °C, (**d**,**d’**) 1000 °C, (**e**,**e’**) 1200 °C, (**f**,**f’**) 1400 °C, and (**g**,**g’**) 1500 °C.

Figure 9 illustrates the surface morphology of the S2 samples subjected to thermal shock at several temperature differences. The images, labeled (a) through (g), represent the S2 after thermal shock at room temperature, 600 °C, 800 °C, 1000 °C, 1200 °C, 1400 °C, and 1500 °C, respectively. At a temperature difference of 600 °C, the surface remains largely unchanged from its pre-shock state, retaining its smooth texture devoid of oxidation marks and significant pores. This suggests minimal oxidation at lower temperatures, preventing evident pore formation (Figure 9b,b’). Upon reaching 800 °C, a thin oxidation layer emerges on the surface, signaling that the S2 sample experienced some oxidation during this phase. The surface exhibits smaller pores, decreased compactness, and a rougher texture than before the shock, aligning with previous observations of the material’s altered residual flexural strength. The presence of these pores not only compromises the sample’s density but could also lead to micro-crack development under lower stress levels, undermining its mechanical robustness (Figure 9c,c’).

As the thermal shock temperature reaches 1000 °C, the surface turns notably rougher and more porous, with a substantial increase in fragmented oxidation layer pieces. The surface pore count declines due to surface BN and Si${}_{3}$N${}_{4}$ reacting with ambient oxygen, creating a liquid oxide that coats the material surface. This oxide layer bridges any existing micro-cracks and pores while also providing a protective barrier against thermal stress during quenching (Figure 9d,d’). Consequently, the ceramics demonstrate enhanced residual flexural strength after this level of thermal shock.

**Figure 10:**
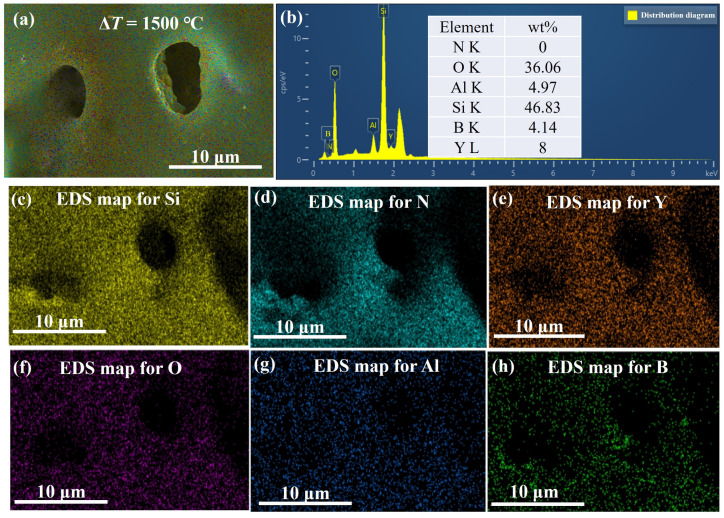
EDS analysis and elemental content of the surface of S2 samples after 1500 °C thermal shock. (**a**) high magnification, (**b**) EDS analysis and elemental content, elemental distribution of (**c**) Si, (**d**) N, (**e**) Y, (**f**) O, (**g**) Al, and (**h**) B.

**Figure 11:**
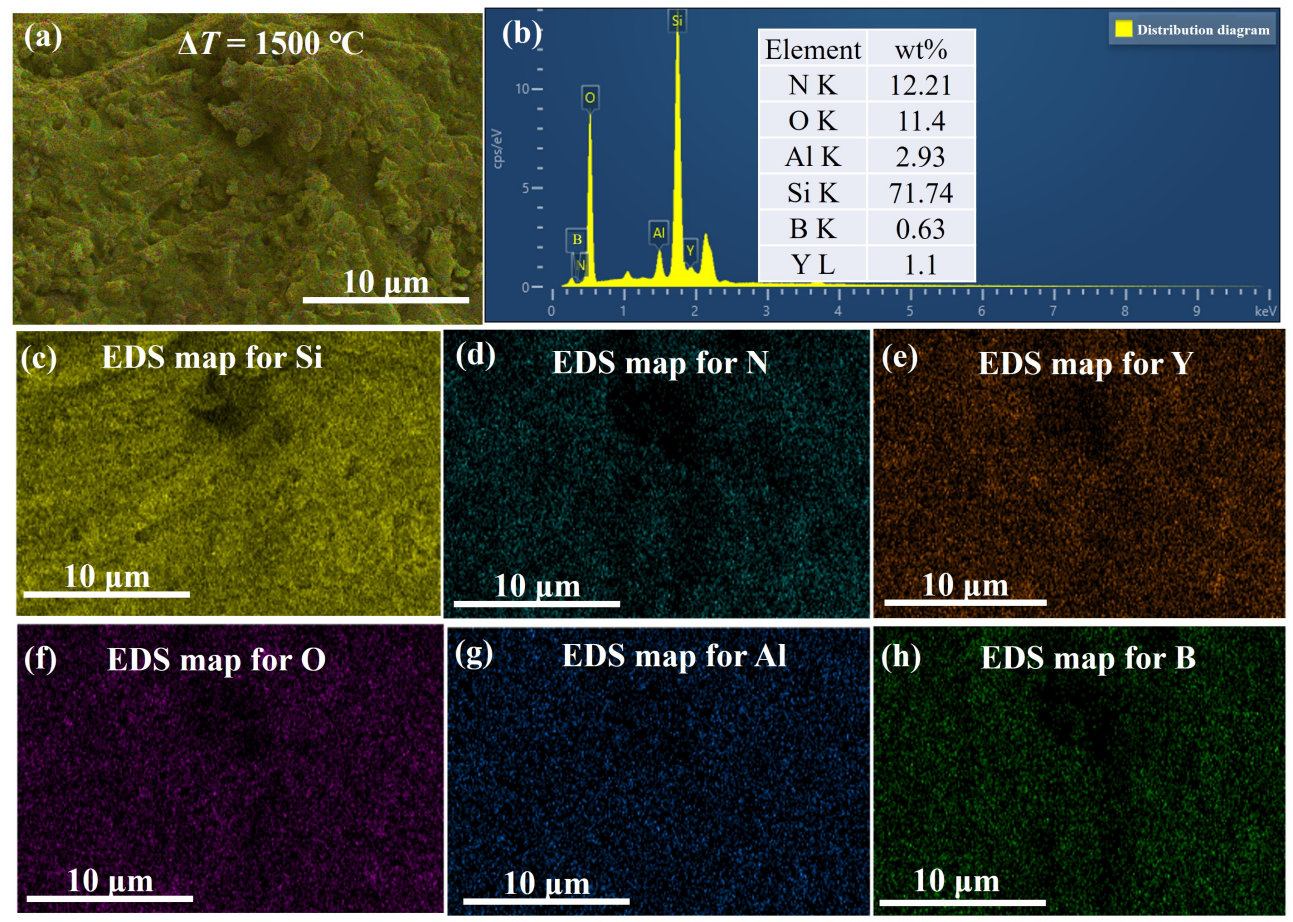
EDS analysis and elemental content of the fracture of S2 samples after 1500 °C thermal shock. (**a**) high magnification, (**b**) EDS analysis and elemental content, elemental distribution of (**c**) Si, (**d**) N, (**e**) Y, (**f**) O, (**g**) Al, and (**h**) B.

The 1200 °C thermal shock yields a sintered sample surface that is smooth and devoid of discernible pores or micro-cracks but with a pronounced oxidation film. At 1400 °C, the surface exhibits pores approximately 2um wide, which disrupt the oxide film’s continuity. Numerous barb-like formations are visible on the surface, and some samples even show fractures. Finally, at 1500 °C, the surface becomes extremely coarse with many burst bubbles. Figure 10 and Figure 11 show that this coarseness results from the formation of a highly fluid phase in the Y${}_{2}$O${}_{3}$-Al${}_{2}$O${}_{3}$-B${}_{2}$O${}_{3}$ glass ceramic system. Once the BN oxidizes, oxygen breaches the low-viscosity Y${}_{2}$O${}_{3}$-Al${}_{2}$O${}_{3}$-B${}_{2}$O${}_{3}$ protective layer and reacts further with the underlying BN, yielding B${}_{2}$O${}_{3}$ and N${}_{2}$. Both products, highly volatile at this temperature, evaporate from inside the sample.

### 3.5. Fracture Morphology

**Figure 12:**
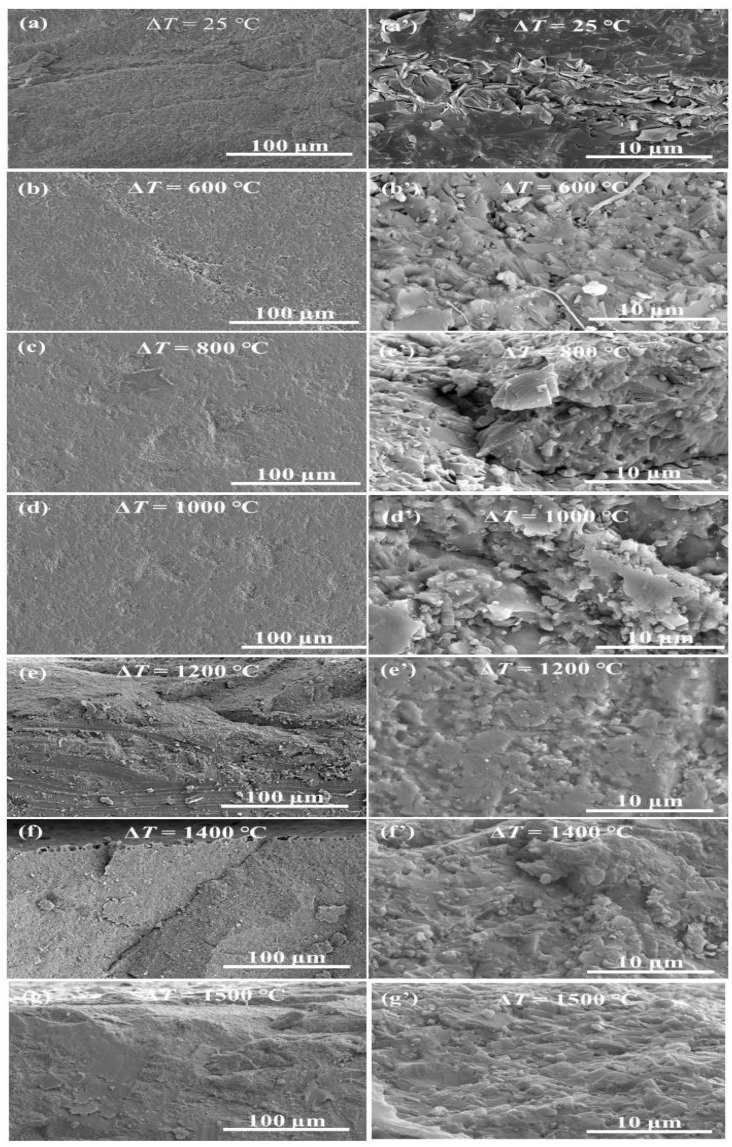
SEM micrographs of fracture surfaces of Si${}_{3}$N${}_{4}$/BN fibrous monolithic ceramics after thermal shock at several temperature differences: (**a**,**a’**) 25 °C, (**b**,**b’**) 600 °C, (**c**,**c’**) 800 °C, (**d**,**d’**) 1000 °C, (**e**,**e’**) 1200 °C, (**f**,**f’**) 1400 °C, and (**g**,**g’**) 1500 °C.

Figure 12 illustrates the fracture morphology of the S2 samples after thermal shock at several temperature differences. Upon examining the post thermal shock fracture morphology of these ceramics, it is evident that the samples exposed to thermal shocks exhibit new cracks, pores, and other defects. These defects arise due to the thermal stresses experienced during the thermal shock process, leading to subsequent damage.

The central fracture zone, when juxtaposed with fractures observed at room temperature, shows minimal alterations following shocks at multiple temperature points. The fracture behavior of the ceramic remains consistent, characterized by its inherent brittle nature. This surface appears irregular and coarse, devoid of any discernible cracks. Concurrently, upon inspecting the periphery of the fracture and the surface contact region of the S2 sample post thermal shock, a distinct oxidation layer becomes evident. This layer’s thickness augments with the intensity of the thermal shock. Specifically, at 1200 °C, a slender oxidation layer coats the S2 ceramic material’s surface. As temperature fluctuations surpass 1200 °C, the oxidation layer thickens progressively. Moreover, the oxidation extent intensifies in direct proportion to the increase in thermal shock temperature variance.

Building on Griffith’s theory, a material subjected to external force induces a stress concentration at the crack tip, as defined in Equation (1) [1]. Herein:

(3) $$\sigma_{A}=\sigma \left(1+2\sqrt{c/\rho }\right),$$

$\sigma_{A}$ is the stress concentration at the crack tip, σdenotes the externally applied stress, c is the crack’s length, ρ is the curvature radius at the crack tip. When a crack is present, the stress at its tip vastly supersedes the overall external stress that the material sustains. If this localized stress escalates due to the increased external load, approaching the material’s fracture threshold, the crack will proliferate rapidly, culminating in the material’s comprehensive fracture. Upon examining the multitude of defects within the composite ceramics post thermal shock, it is clear that the unexpected surge in the ceramics’ residual strength post-experimentation diverges from predictions rooted in classical fracture mechanics. Consequently, the predominant factor bolstering residual strength can be attributed to the transformative behavior of the composite ceramics’ surface microstructure during the thermal shock phase.

## 4. Conclusions

The manuscript delves into the thermal shock resistance of Si${}_{3}$N${}_{4}$/BN fibrous monolithic ceramics, exploring the underlying mechanisms of thermal shock damage and suggesting effective techniques to enhance the resistance of composite ceramics. The primary findings are as follows:

1. Residual flexural strength analysis: the flexural strength of Si${}_{3}$N${}_{4}$/BN fibrous monolithic ceramics first declines and then rises with an increasing thermal shock temperature differential. For thermal shock temperature differences below 800 °C, the material’s residual flexural strength reduces proportionally. However, when this difference surpasses 1000 °C, the strength begins to rise. Notably, all samples achieved their optimal residual strength after exposure to a 1500 °C temperature differential;
2. BN concentration influence: When the BN mass fraction is 5 wt.%, the residual strength after a thermal shock at a temperature difference of 1500 °C is 387 ± 19 MPa, which is 124% higher than the original strength of the sample that did not undergo thermal shock (25 °C, 311 ± 18 MPa);
3. Oxide film in oxygen-rich environments: When Si${}_{3}$N${}_{4}$/BN ceramics encounter an oxygen-abundant atmosphere during thermal shocks, they generate a compact Y${}_{2}$O${}_{3}$-Al${}_{2}$O${}_{3}$-B${}_{2}$O${}_{3}$ oxide film on their surface, which significantly augments the ceramic’s thermal shock resistance.

## Figures and Tables

**Table 1:** The compositional components of the silicon nitride fibers.

α-Si${}_{3}$N${}_{4}$ (wt.%)	(wt.%)	Al${}_{2}$O${}_{3}$ (wt.%)	Deionized Water (wt.%)	(C${}_{6}$H${}_{7}$N${}_{a}$O${}_{6}$)${}_{x}$ (wt.%)
47.5	1.78	0.72	48.75	1.25

**Table 2:** Parameters of the mass fraction of BN in Si${}_{3}$N${}_{4}$/BN fibrous monolithic ceramics.

Composites	BN ( wt.%)
S1	3
S2	5
S3	10
S4	50

**Table 3:** The relative density and apparent porosity of Si${}_{3}$N${}_{4}$/BN fibrous monolithic ceramics.

Composites	Theoretical Density g·cm${}^{-3}$	Actual Density g·cm${}^{-3}$	Relative Density %	Apparent Porosity %
S1	3.33	3.27	98.3	0.93
S2	3.26	3.15	96.8	1.25
S3	3.17	2.96	93.5	1.88
S4	2.67	2.24	83.8	3.52

## Data Availability

Not applicable.
